# The AMPK inhibitor overcomes the paradoxical effect of RAF inhibitors through blocking phospho–Ser-621 in the C terminus of CRAF

**DOI:** 10.1074/jbc.RA118.004597

**Published:** 2018-07-20

**Authors:** Jimin Yuan, Wan Hwa Ng, Jiajun Yap, Brandon Chia, Xuchao Huang, Mei Wang, Jiancheng Hu

**Affiliations:** From the ‡ Division of Cellular and Molecular Research, National Cancer Centre Singapore, 11 Hospital Drive, Singapore 169610, Singapore and; the § Cancer and Stem Cell Program, Duke-NUS Medical School, 8 College Road, Singapore 169857, Singapore

**Keywords:** cancer, cancer therapy, cell signaling, drug resistance, cancer biology, 14-3-3, AMP-activated protein kinase, paradoxical activation, RAF inhibitor, RAF kinase

## Abstract

The dimerization-driven paradoxical activation of RAF proto-oncogene Ser/Thr kinase (RAF) is the predominant cause of drug resistance and toxicity in cancer therapies with RAF inhibitors. The scaffold protein 14-3-3, which binds to the RAF C terminus, is essential for RAF activation under physiological conditions, but the molecular basis is unclear. Here we investigated whether and how 14-3-3 regulates the dimerization-driven transactivation of the RAF isoform CRAF by RAF inhibitors and affects drug resistance and toxicity by virtue of the dominant role of CRAF in these processes. We demonstrated that 14-3-3 enhances the dimerization-driven transactivation of CRAF by stabilizing CRAF dimers. Further, we identified AMP-activated protein kinase (AMPK) and CRAF itself as two putative kinases that redundantly phosphorylate CRAF's C terminus and thereby control its association with 14-3-3. Next, we determined whether the combinatory inhibition of AMPK and CRAF could overcome the paradoxical effect of RAF inhibitors. We found that the AMPK inhibitor (AMPKi) not only blocked the RAF inhibitor–driven paradoxical activation of ERK signaling and cellular overgrowth in Ras-mutated cancer cells by blocking phosphorylation of Ser-621 in CRAF but also reduced the formation of drug-resistant clones of BRAF^V600E^-mutated cancer cells. Last, we investigated whether 14-3-3 binding to the C terminus of CRAF is required for CRAF catalytic activity and observed that it was dispensable *in vivo*. Altogether, our study unravels the molecular mechanism by which 14-3-3 regulates dimerization-driven RAF activation and identified AMPKi as a potential agent to counteract drug resistance and adverse effects of RAF inhibitors in cancer therapies.

## Introduction

Ras–RAF–MEK–ERK^2^ signaling plays a central role in cell proliferation, survival, and differentiation (1–4). In normal cells, this signaling cascade is tightly regulated, and its hyperactivation causes human cancers and developmental disorders. The Ser/Thr protein kinase RAF is a core component of this signaling cascade and includes three isoforms: BRAF, CRAF, and ARAF (5–7). All RAF isoforms have similar molecular structures with distinct traits that result in their differential ability to activate their downstream effector, MEK (8–11). Recent studies have identified RAF dimerization as a key event in the activation and regulation of this signaling cascade (12–17). In a RAF dimer, the catalysis-deficient protomer, either because of mutation or inhibitor loading, facilitates the other protomer to assemble in an active conformation and thus triggers its catalytic activity (8). RAF dimerization not only contributes to the activation of RAF–MEK–ERK signaling under physiological and pathological conditions but is also one of the important mechanisms that underlie RAF inhibitor resistance in cancer therapy (18–20). Blockage of dimerization-driven transactivation of RAF kinase has important implications in RAF inhibitor–mediated cancer therapy.

Dimerization-driven transactivation of RAF kinase is regulated by many factors. Canonical upstream Ras activation has been shown to facilitate RAF dimerization, probably through relieving the intramolecular interaction between the Ras-binding domain and kinase domain (14). Similarly, RAF inhibitors promote RAF dimerization by altering the conformation of its kinase domain (4). The dimerization-favored conformation of the RAF molecule could be also achieved by catalytic spine mutations or β3-αC loop deletions that exist in cancer genomes (11, 21). Furthermore, the differential molecular traits among RAF isoforms result in their distinct propensities in dimerization-driven transactivation. ARAF has a noncanonical APE motif that weakens its dimerization ability and accounts for its lesser activity toward MEK (11). The unique N-terminal acidic (NtA) motif of BRAF enables this isoform to strongly transactivate the other two isoforms through dimerization, whereas the transactivation ability of CRAF and ARAF is regulated by their NtA motif phosphorylation (8). In addition, 14-3-3 has been suggested to regulate the dimer-dependent activation of RAF kinase, but the molecular basis of this regulation remains unknown (14, 22).

14-3-3 is a family of dimeric scaffold proteins that bind to phospho-Ser/Thr within RS*X*pS/TXP or R*XXX*pS/T*X*P motifs (23–25). All RAF isoforms contain a conserved 14-3-3 binding motif in their C terminus, although which kinase(s) is/are responsible for its phosphorylation remains controversial (26–29). Because CRAF is the key isoform of RAF kinase responsible for the RAF inhibitor-induced paradoxical activation of RAF–MEK–ERK signaling in cancer therapy, in this study, we used it as a module to explore the molecular mechanism that governs 14-3-3 binding to the C terminus of RAF kinase and thus regulates its dimerization-driven transactivation. Our data indicated that 14-3-3 binding to the C terminus of CRAF, which is phosphorylated redundantly by AMPK and CRAF itself, facilitates CRAF transactivation by enhancing its dimerization. Furthermore, we evaluated the potential value of the AMPKi against the paradoxical effect of RAF inhibitors in cancer therapy and demonstrated that the AMPKi could not only effectively block RAF inhibitor–induced activation of RAF–MEK–ERK signaling and cellular overgrowth in Ras-mutated cancer cells but also reduce the formation of drug-resistant clones derived from BRAF^V600E^-mutated cancer cells. To extend our findings, we investigated the role of 14-3-3 in the catalysis of active CRAF and found that it is dispensable for CRAF catalytic activity *in vivo*. Taken together, this study elucidates how 14-3-3 regulates the dimerization-driven transactivation of RAF kinase and provides a potential approach to overcome the resistance and adverse effects of RAF inhibitors in cancer therapy.

## Results

### 14-3-3 binding to the C terminus of CRAF regulates its dimerization-driven transactivation by enhancing its dimerization

**Figure 1.**
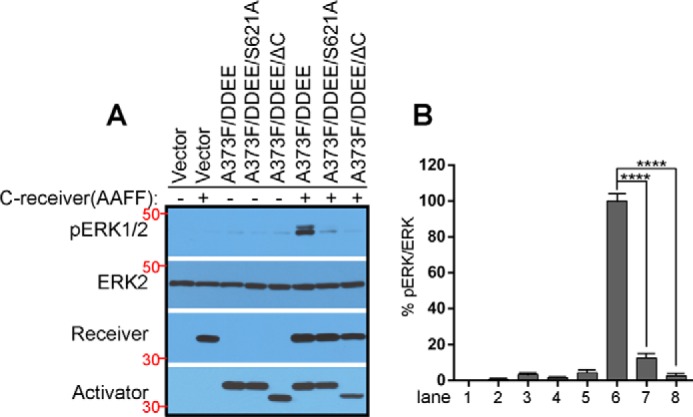
**Disruption of 14-3-3 binding to the C terminus of CRAF blocks the dimerization-driven transactivation of CRAF.** *A* and *B*, the CRAF co-activation assay was carried out as described before (8, 21). Briefly, different allosteric CRAF mutants were individually co-expressed with the CRAF receiver (CKD/AAFF, aa 322–648) in 293T cells, and the activation of ERK1/2 was measured by anti-phospho-ERK1/2 immunoblot (*A*). Phospho-ERK1/2 and ERK2 were quantified by ImageJ, and their ratio was calculated (*B*) (*n* = 3; ****, *p* < 0.0001). All images are representative of at least three independent experiments.

Previous studies have suggested that 14-3-3 binding to the C terminus of RAF kinase is critical for its dimer-dependent activation (14, 22). To confirm this finding and further explore the molecular details of this regulation, we deleted or mutated the C-terminal 14-3-3 binding motif of the allosteric CRAF mutant that has a fused catalytic spine and an acidic NtA motif (DDEE/A373F) (30, 31). Using these kinase-dead mutants (DDEE/A373F/S621A and DDEE/A373F/ΔC), we carried out an RAF co-activation assay developed in our previous studies (8, 21). When co-expressed in 293T cells, these mutants had much less ability to transactivate the WT CRAF receiver and, in turn, induced less phosphorylation of ERK1/2 in contrast to their prototype (Fig. 1, *A*, *seventh* and *eighth lanes*, and *B*). These data demonstrated that 14-3-3 binding to the C terminus of CRAF indeed plays a critical role in the dimerization-driven transactivation of CRAF.

**Figure 2.**
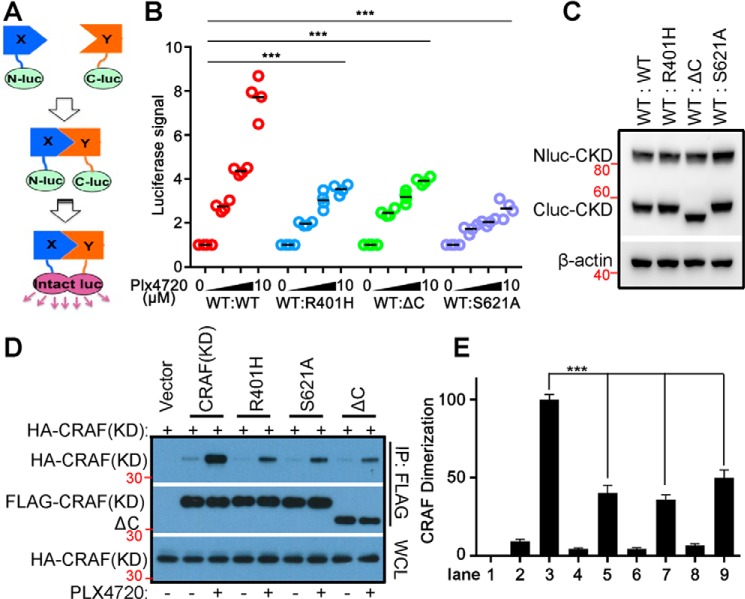
**Disruption of 14-3-3 binding to the C terminus of CRAF inhibits RAF inhibitor–induced dimerization.** *A–C*, the dimer affinity of CRAF or its mutants was measured by complimentary split luciferase assay as detailed under “Experimental procedures.” *A*, a diagram illustrating the complimentary split luciferase assay. *B*, luciferase signals were measured to study the interaction between WT and mutant CRAFs (aa 322–648; R401H, S621A, and ΔC). Briefly, 293T cells co-expressing Nluc- or Cluc-fused CRAF and its mutants were treated with PLX4720 before the luciferase signals were measured using an illuminometer. Each dot represents the means from the technical repeat of triple wells in the same study (*n* = 4; ***, *p* < 0.001). *C*, the protein level of Nluc- and Cluc-CRAF fusion proteins in 293T cells from *B* was measured by immunoblot. *D* and *E*, the dimerization of CRAF or its mutants was evaluated by co-immunoprecipitation assay. HA-tagged CKD (aa 322–648) was co-expressed with FLAG-tagged CKD or its mutants in 293T cells. Then cells were treated with or without 10 μm PLX4720 for 2 h, and immunoprecipitation was carried out with anti-FLAG beads and lysis/washing buffer containing 0.1% NP-40 with or without 10 μm PLX4720. The HA- and FLAG-tagged CKD or mutants in immunoprecipitants were detected by immunoblots (*D*) and quantified by ImageJ, and their ratio was calculated (*E*) (*n* = 3; ***, *p* < 0.001). All images are representative of at least three independent experiments.

To understand how 14-3-3 regulates the dimerization-driven transactivation of CRAF, we measured the dimer affinity of CRAF mutants with either deletion or mutation of the C-terminal 14-3-3 binding motif by complementary split luciferase assays (Fig. 2*A*). To do this, Nluc (the N terminus of luciferase) and Cluc (the C terminus of luciferase) were fused, respectively, to the CRAF kinase domain (CKD, aa 324–648) with or without the S621A mutation or deletion of the 14-3-3 binding motif (hereafter referred to as ΔC). Then Nluc-CKD was co-expressed with either Cluc-CKD/S621A or Cluc-CKDΔC in 293T cells (Fig. 2*B*). The Cluc-fused R401H mutant (Cluc-CKD/R401H), which has a disrupted dimer interface, was used as a control. The RAF inhibitor PLX4720 (vemurafenib) induced much less luciferase signaling in 293T transfectants that express either Cluc-CKD/S621A, Cluc-CKD ΔC, or Cluc-CKD/R401H, in contrast to those expressing the WT counterpart (Fig. 2*C*), suggesting that CKD/S621A and CKDΔC have much less ability than CKD/R401H to associate with CKD upon inhibitor loading. To further confirm this finding, we next carried out a co-immunoprecipitation assay. As shown in Fig. 2, *D* and *E*, treatment of 293 transfectants that express CKD with PLX4720 induced robust dimerization that is impaired by S621A mutation or deletion of the 14-3-3 binding motif (ΔC) as was done by R401H mutation. Taken together, these data indicate that 14-3-3 binding to the C terminus of CRAF improves the dimerization-driven transactivation of CRAF by facilitating its dimerization or stabilizing its dimers.

### The C-terminal 14-3-3 binding motif of CRAF is phosphorylated redundantly by AMPK and CRAF itself, which is essential for the association of 14-3-3 with CRAF

**Figure 3.**
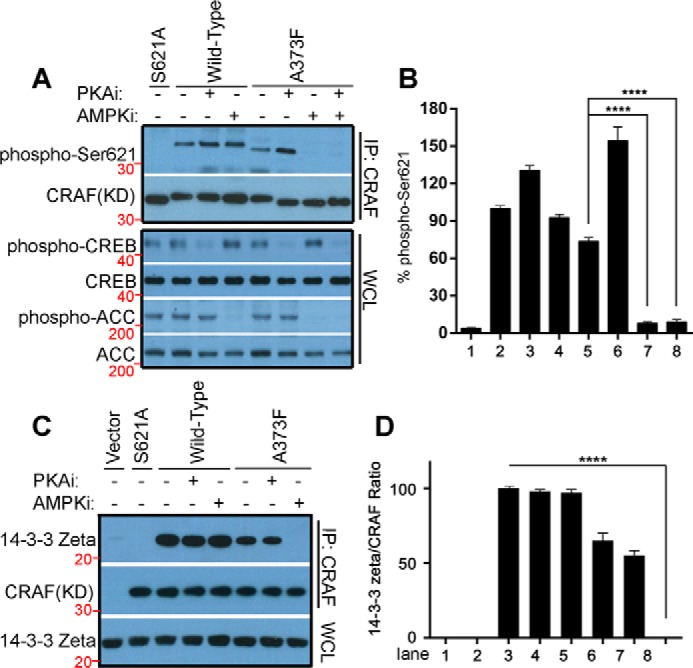
**Ser-621 in the 14-3-3 binding motif of CRAF is redundantly phosphorylated by AMPK and CRAF itself, but not by PKA, which is essential for 14-3-3 association.** The WT or the kinase-dead mutant (A373F) of CRAF (aa 322–648) was expressed in 293T cells and treated with the indicated inhibitors (10 μm H89, 5 μm Compound C) for 4 h before lysis for immunoprecipitation. *A* and *B*, the phospho–Ser-621 of CRAF and its mutants was detected by immunoblot (*A*) and quantified using ImageJ (*B*) (*n* = 3; ****, *p* < 0.0001). The activity of PKA and AMPK was probed as phospho-CREB or phospho-ACC in whole-cell lysates, respectively (*A*, *bottom panel*). *C*, the association of CRAF or its mutants with 14-3-3 in *A* was examined by immunoprecipitation and immunoblot. *D*, the signals of 14-3-3 and CRAF in immunoprecipitants were quantified by ImageJ, and their ration was calculated (*D*) (*n* = 3; ****, *p* < 0.0001). All images are representative of at least three independent experiments.

It is well-known that the binding of 14-3-3 to the C terminus of CRAF requires the phosphorylation of Ser-621 in the RS*X*S*X*P motif. Previous studies have suggested that the phosphorylation of Ser-621 is mediated by PKA, AMPK, or CRAF in different contexts (26–28). To clarify which kinase(s) target(s) this site and thus regulates the dimerization-driven transactivation of CRAF, we examined phospho–Ser-621 of WT CRAF or its kinase-dead mutant (A373F) expressed in 293T cells with or without pharmaceutical inhibition of PKA or AMPK. As shown in Fig. 3*A*, *bottom panel*, treatment of 293 transfectants with the PKA inhibitor H89 dramatically diminished the phosphorylation of cAMP response element–binding protein (CREB), a direct target of PKA, whereas treatment with the AMPK inhibitor Compound C completely blocked the phosphorylation of acetyl-CoA carboxylase (ACC), a putative effector of AMPK, suggesting that the activity of PKA or AMPK, respectively, is inhibited in these cells. However, only the kinase-dead A373F mutant lost phospho–Ser-621 upon AMPK inhibition with Compound C (Fig. 3*A*, *seventh lane*), whereas PKA inhibition with H89 slightly enhanced phospho–Ser-621 in both WT and kinase-dead A373F CRAF (Fig. 3*A*, *third* and *sixth lanes*). These data indicated that Ser-621 in the 14-3-3 binding motif of CRAF is phosphorylated redundantly by AMPK and CRAF itself, although the mechanism for the slight increase of phospho–Ser-621 in the presence of the PKA inhibitor is unclear and requires further investigation in the future. Further, we demonstrated by co-immunoprecipitation assay that the blockage of phospho–Ser-621 in kinase-dead A373F CRAF by Compound C impaired the association of 14-3-3 with this mutant (Fig. 3, *C* and *D*).

### AMPKi blocks the paradoxical stimulation of RAF–MEK–ERK signaling and cell growth by RAF inhibitors in Ras-mutated cancer cells

**Figure 4.**
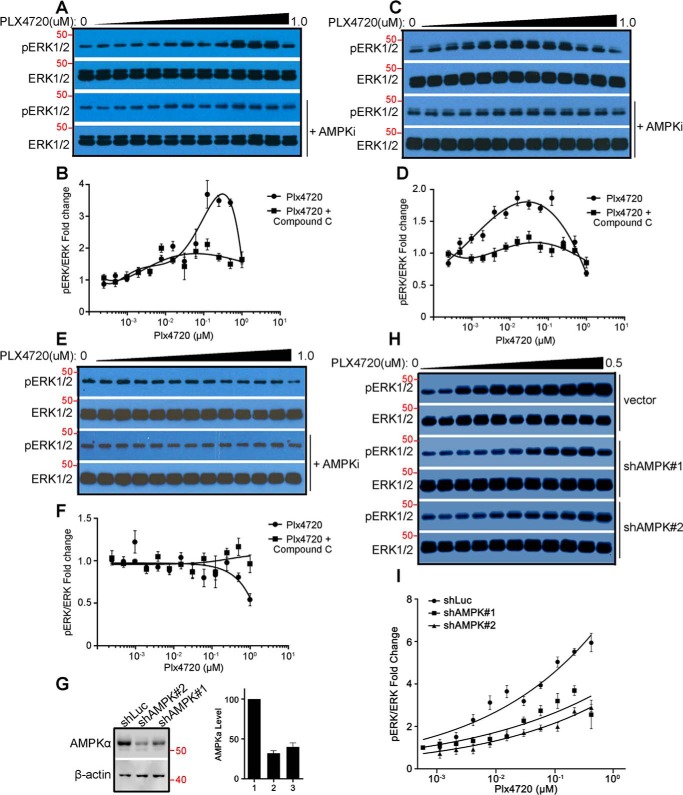
**The AMPKi blocks the paradoxical activation of RAF–MEK–ERK signaling by the RAF inhibitor.** *A–F*, the AMPK inhibitor Compound C abolishes the paradoxical activation of RAF–MEK–ERK signaling by the RAF inhibitor PLX4720. H1299 lung cancer cells (Nras and Q61R, *A* and *B*), Sk-mel-2 melanoma cancer cells (Nras and Q61R, *C* and *D*), and H522 lung cancer cells (WT Ras, *E* and *F*) were treated by gradually increasing concentrations of PLX4720 with or without concurrent treatment of low doses of Compound C. The phospho-ERK1/2 and ERK1/2 were detected by immunoblots (*A*, *C*, and *E*). The concentrations of Compound C were 0.16 μm, 1.0 μm, and 0.63 μm for H1299, Sk-mel-1, and H522 cells respectively. The dose–response graphs for the ratios of phospho-ERK1/2 *versus* total ERK1/2 measured from *A*, *C*, and *E* are plotted in *B*, *D*, and *F* accordingly. *G–I*, AMPKα knockdown impairs the paradoxical activation of RAF–MEK–ERK signaling by RAF inhibitor PLX4720. The expression of AMPKα in H1299 stable cell lines with shRNAs against human AMPKα1 or luciferase was detected by immunoblot and quantified by ImageJ (*G*). *H* and *I*, the response of these cell lines to PLX4720 was examined and graphed as in *A* and *B*. All images are representative of at least three independent experiments.

**Figure 5.**
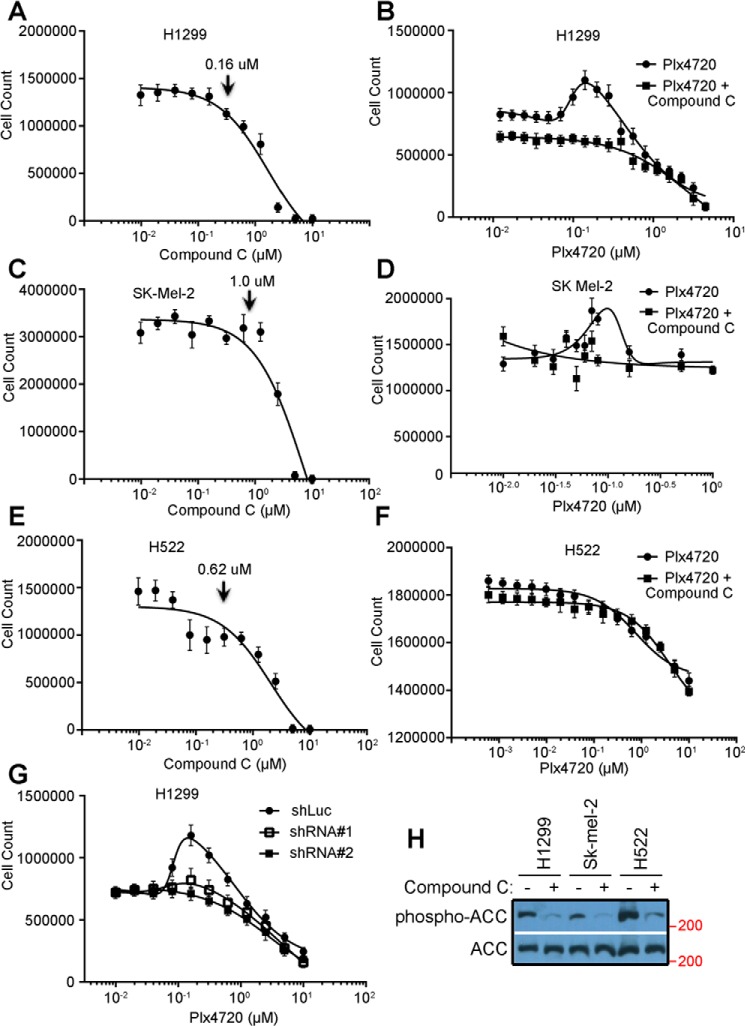
**The AMPKi inhibits the enhanced proliferation of Ras-mutated cancer cell lines induced by the RAF inhibitor.** *A*, *C*, and *E*, the nontoxic concentrations of Compound C for different cancer cell lines were determined by cell growth assays in medium with 2-fold diluted drug. H1299 lung cancer cells, Sk-mel-2 melanoma cancer cells, and H522 lung cancer cells were tolerant to 0.16 μm, 1.0 μm, and 0.62 μm Compound C, respectively. *B*, *D*, and *F*, the cell proliferation profiles in medium with 2-fold diluted PLX4720 with or without Compound C at the tolerant concentrations (0.16 μm for H1299, 1.0 μm for Sk-mel-2, or 0.62 μm for H522) were constructed by cell counting after 1-week culture. *G*, the cell proliferation profiles of H1299 stable cell lines with shRNA against AMPKα1 or luciferase were also obtained as in *B. H*, the inhibition of AMPK activity in cancer cells treated with Compound C at the tolerant concentrations in *B*, *D*, and *F* was confirmed by anti-phospho-ACC immunoblot. All images are representative of at least three independent experiments.

The paradoxical activation of RAF–MEK–ERK signaling driven by RAF inhibitors is not only responsible for the intrinsic resistance of Ras-mutated cancers but also one of the important causes that lead to acquired resistance in BRAF^V600E^-harboring cancers (32). Moreover, CRAF has been shown to be a key isoform of RAF kinase that mediates RAF inhibitor–induced paradoxical activation of this signaling pathway (18–20). As it has been demonstrated that the dimerization-driven transactivation of CRAF requires phosphorylation of the C-terminal 14-3-3 binding motif redundantly by AMPK and CRAF itself, we next investigated whether AMPKi blocks the RAF inhibitor–induced paradoxical activation of RAF–MEK–ERK signaling in Ras-mutated cancer cells, thus representing a viable combination strategy. As reported before, the RAF inhibitor vemurafenib activated RAF–MEK–ERK signaling in a paradoxical manner in the Ras-mutated cancer cell lines H1299 (NrasQ61K) and Sk-mel-2 (NrasQ61R) but not in the Ras-WT cancer cell line H522 (Fig. 4, *A–F*). This paradoxical effect of vemurafenib on H1299 and Sk-mel-2 cancer cell lines was blocked by the AMPK inhibitor Compound C (Fig. 4, *A–F*) or the shRNA-mediated knockdowns of AMPKα1, the predominant isoform of AMPKα in H1299 cells (Fig. 4, *G–I*). Furthermore, the paradoxical stimulation of cell growth by vemurafenib in H1299 and Sk-mel-2 cancer cell lines was also inhibited by Compound C or AMPKα1 knockdown (Fig. 5, *A*–G). Noteworthy, the concentrations of Compound C used in our experiments could effectively block the phosphorylation of ACC by AMPK (Fig. 5*H*) but had no apparent toxicity in these cell lines (Fig. 5, *A*, *C*, and *E*), which excludes the potential artifacts arising from this AMPK inhibitor. Altogether, these data further support that AMPK- and CRAF-mediated phosphorylation of the C-terminal 14-3-3 binding motif plays a critical role in the dimerization-driven transactivation of CRAF.

### AMPKi reduces the drug-resistant clones derived from BRAF^V600E^-harboring cancer cell lines

**Figure 6.**
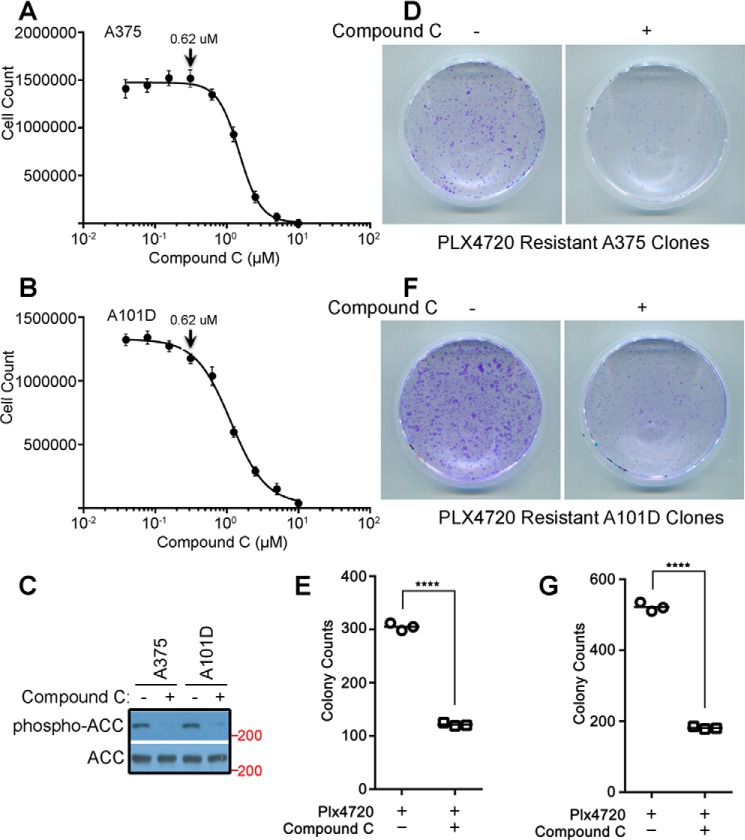
**The AMPKi reduces the formation of RAF inhibitor-resistant clones derived from BRAF^V600E^-harboring cancer cells.** The nontoxic concentrations of Compound C in A375 and A101D melanoma cell lines were determined as in Fig. 5. *A–C*, both cell lines were tolerant to 0.62 μm Compound C (*A* and *B*), which effectively inhibits the AMPK activity, indicated by a dramatically decreased phospho-ACC signal in the immunoblot (*C*). *D–G*, the PLX4720-resistant clones were generated, respectively, from A375 and A101D in cultures with or without 0.62 μm Compound C as described under “Experimental Procedures,” stained with crystal violet (*D* and *F*), and counted manually (*E* and *G*) (*n* = 3; ****, *p* < 0.0001). All images are representative of at least three independent experiments.

As described above, the paradoxical activation of RAF–MEK–ERK signaling contributes significantly to acquired resistance in the treatment of BRAF^V600E^-harboring cancers with RAF inhibitors. Hence we examined whether AMPKi would enhance the efficacy of RAF inhibitors by impairing the drug resistance in BRAF^V600E^-harboring cancers. To this end, we treated A375 and A101D, two BRAF^V600E^-positive melanoma cell lines, with vemurafenib alone or plus Compound C and identified the formation of drug-resistant clones by crystal violet staining. As shown in Fig. 6, the addition of Compound C at a concentration without apparent toxicity (0.62 μm) effectively blocked the phosphorylation of ACC by AMPK and dramatically reduced the formation of drug-resistant clones from both melanoma cell lines.

### The 14-3-3 binding to the C terminus of constitutively active CRAF R-spine mutant elevates its dimer affinity, which is required for in vitro but not in vivo catalytic activity

**Figure 7.**
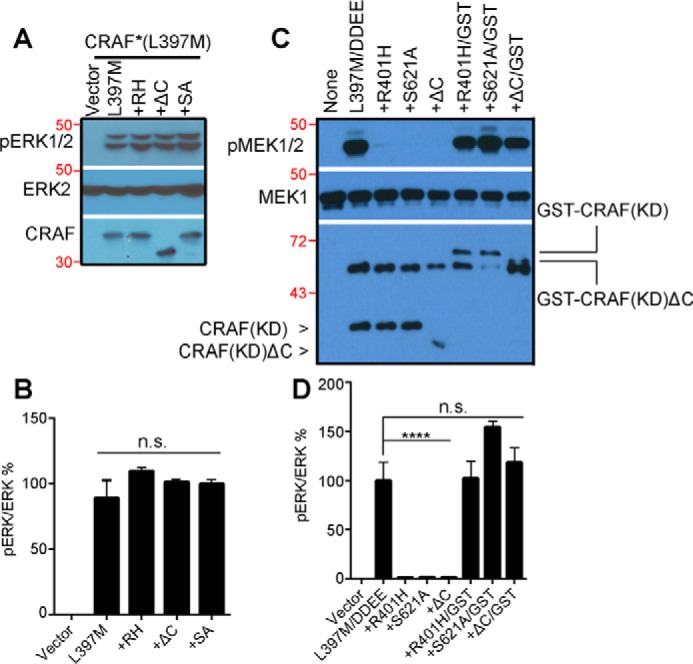
**14-3-3 binding to the C terminus of constitutively active CRAF mutants is not required for *in vivo* activity but for *in vitro* catalytic activity.** *A*, the S621A mutation or deletion of the C-terminal 14-3-3 binding motif does not affect the activity of the CRAF R-spine mutant *in vivo*. CRAF mutants were expressed in 293T cells, and their activity was measured by anti-phospho-ERK1/2 immunoblot. *B*, the ratio quantification of phospho-ERK1/2 *versus* ERK2 in *A* (*n* = 3; *n.s.*, not significant). *C*, active CRAF R-spine mutants with S621A mutation or deletion of the C-terminal 14-3-3 binding motif lose their activity *in vitro* upon purification, which is rescued by GST fusion. The CRAF mutants in *A* were purified by immunoprecipitation, and their activity was measured by *in vitro* kinase assay. *D*, the ratio quantification of phospho-MEK1/2 *versus* MEK1/2 in *C* (*n* = 3; ****, *p* < 0.0001). All images are representative of at least three independent experiments.

Dimerization of RAF kinase is required for both activation and catalytic activity (11, 33). Our recent study has shown that even constitutively active RAF mutants function as dimers to phosphorylate MEK, and mutants with a low dimer affinity lose their catalytic activity *in vitro* upon purification, which can be restored by GST fusion–enhanced dimerization (11). In this study, we have demonstrated that 14-3-3 binding to the C terminus of CRAF is required for its dimerization-driven transactivation. However, whether it regulates the catalytic activity of CRAF after activation remains unknown. To address this question, we introduced the S621A mutation or deleted the C-terminal 14-3-3 binding motif in the constitutively active regulatory spine mutant of CRAF (CRAF/DDEE/L397M) generated in our previous studies (21, 34) and examined whether these alterations would impair its catalytic activity. As shown in Fig. 7, *A* and *B*, CRAF/DDEE/L397M/S621A and CRAF/DDEE/L397M/ΔC mutants exhibited activity as high as that of their parental protein when expressed in 293T cells. However, resembling the constitutively active CRAF/DDEE/L397M/R401H mutant with the altered dimer interface (11), these mutants lost their catalytic activity *in vitro* upon purification, which is rescued by GST fusion (Fig. 7, *C* and *D*). These data suggest that the CRAF/DDEE/L397M/S621A and CRAF/DDEE/L397M/ΔC mutants have a lower dimer affinity than their parental protein, although they do not associate with 14-3-3, and further support that 14-3-3 binding to the C terminus of CRAF elevates its dimer affinity or stabilizes CRAF dimers.

## Discussion

Dimerization-driven transactivation plays a pivotal role in regulating the activity of RAF kinase under variable physiology and pathology conditions (35). Kinase-dead mutants or inhibitor-bound RAF molecules turn on their WT counterparts through this mechanism and induce hyperactive RAF–MEK–ERK signaling, which alters cellular functions. The dimerization-driven transactivation of RAF kinase is regulated by distinct factors, such as the scaffold protein 14-3-3. It has been speculated that the 14-3-3 dimer binds to the C terminus of the RAF dimer and thus facilitates RAF dimerization (14, 21). This study provides strong evidence supporting this notion. Specifically, we have shown that CRAF mutants that are unable to associate with 14-3-3 have much less dimer affinity and fail to transactivate WT CRAF. Furthermore, although constitutively active CRAF mutants are able to form dimers without 14-3-3 association *in vivo*, these dimers are so weak that they dissociate during purification and thus lose catalytic activity *in vitro*. This is consistent with our recent findings from oncogenic RAF mutants with β3-αC loop deletions that suggest that strong dimerization drives transactivation of RAF kinase, whereas weak dimerization is required for catalytic activity following its activation (11).

14-3-3 binds to the RS*X*S*X*P or R*XXX*S/T*X*P motif only when its Ser/Thr is phosphorylated (23). However, the protein kinase that phosphorylates the14-3-3 binding motif in the C terminus of RAF kinase was not clearly defined prior to this study, despite the potential involvement of PKA, AMPK, or RAF (26–29). Using kinase-dead mutants and pharmaceutical inhibitors, we have demonstrated that the C-terminal 14-3-3 binding motif of the CRAF isoform is redundantly phosphorylated by AMPK and CRAF itself, but not by PKA, in the cell lines we studied. This conclusion is further supported by the finding that AMPK inhibition blocks the paradoxical activation of RAF–MEK–ERK signaling by the RAF inhibitor in cancer cell lines with active Ras mutations.

The paradoxical activation of RAF–MEK–ERK signaling leads to both the intrinsic resistance in Ras-mutated cancers and the acquired resistance in BRAF^V600E^-driven cancers, which severely limits the efficacy of RAF inhibitors in cancer therapy (32). Moreover, it also induces secondary tumors in RAF inhibitor–treated patients. Our finding that AMPK inhibition abolishes the RAF inhibitor–driven paradoxical activation of RAF–MEK–ERK signaling in Ras-mutated cancer cells and thus inhibits their overgrowth provides a potential combination therapy to control Ras mutation–driven cancers. In addition, because combination of the AMPK inhibitor with the RAF inhibitor also dramatically reduces the drug-resistant clones derived from BRAF^V600E^-harboring cancer cells, it will improve the treatment of this type of cancers with RAF inhibitors.

## Experimental procedures

### Chemicals and antibodies

The antibodies used in this study included anti-phospho-ERK1/2 (4370), anti-phospho-MEK1/2 (9154), and anti-MEK1/2 (9124) (Cell Signaling Technology); anti-phospho–Ser-621 (AM00131PU-N, Acris Antibodies); anti-FLAG (F1804, Sigma); anti-HA (4810, Novus Biologicals); anti-ERK2 (SC-154, Santa Cruz Biotechnology); anti-ERK1/2 (A0229, AB Clonal); and horseradish peroxidase–labeled secondary antibodies (31460 and 31430, Invitrogen). PLX4720 (A-1131) was purchased from Active Biochem, H-89 (S1582) and Compound C (S7306) from Selleckchem; and D-luciferin (LUCK-2KG) from Gold Biotechnology. All other chemicals were obtained from Sigma.

### Plasmids, cell lines, and protein expression

All expression vectors used in this study were either purchased from Addgene or synthesized by Integrated DNA Technologies, as noted in the text. All mutants were generated by PCR, tagged with either FLAG or HA or His, and cloned into vectors by Gibson assembly. The pCDNA3.1(+) expression vector (Invitrogen) was used for transient expression in mammalian cells and pET-28a (Novagen) for bacterial expression. To knock down AMPKα, shRNAs that target 5′-GAGGAGAGCTATTTGATTA-3′ or 5′-GCTTGATGCACACATGAAT-3′ in human AMPKα1 were cloned into the lentiviral vector with the U6 promoter by using traditional molecular methods. The shRNA against luciferase (Addgene, 30324) was used as a control.

The cancer cell lines H1299, Sk-mel-2, H522, A375, and A101D were obtained from the ATCC. All cancer cell lines were maintained in Dulbecco's modified Eagle's medium with 10% fetal bovine serum (Hyclone). The AMPKα knockdown H1299 cell lines were constructed by lentiviral transduction as described before (36–38). For exogenous expression, 293T cells were transfected with the appropriate plasmids using the Biotool transfection reagent by following the manufacturer's protocol. His_6_-tagged MEK1 (K97A) was expressed in bacterial BL21(DE3) strains and purified using a nickel column (Qiagen) and following our previous protocol (36).

### Complementary split luciferase assay

293T transfectants that express different pairs of Nluc- and Cluc-fused CRAF or CRAF mutants were plated in 24-well Krystal black image plates at a seeding density of 2 × 10^5^ cells/well. 24 h later, D-luciferin (0.2 mg/ml) and PLX4720 (0, 2.5, 5, and 10 μm) were added to the culture; the incubation was allowed to proceed for 30 min before the luciferase signals were measured by Promega GloMax® Multi Detection System.

### Immunoprecipitation, in vitro kinase assay, and Western blotting

Immunoprecipitations were performed as described previously (8). Briefly, whole-cell lysates were mixed with either anti-HA or anti-FLAG beads (Sigma), rotated in a cold room for 60 min, and washed three times with radioimmune precipitation assay buffer. For *in vitro* kinase assays, the immunoprecipitants were washed once with kinase reaction buffer (25 mm HEPES, 10 mm MgCl_2_, 0.5 mm Na_3_VO_4_, and 0.5 mm DTT (pH 7.4)) and then incubated with 20 μl of kinase reaction mixture (2 μg of substrate and 100 mm ATP in 20 μl of kinase reaction buffer) per sample at room temperature for 10 min. The kinase reaction was stopped by adding 5 μl/sample of 5× Laemmli sample buffer. Immunoblotting was carried out as described before (37). All blots were quantified using ImageJ, and the graphs were generated using GraphPad Prism 6.

### Drug toxicity test and cell proliferation assay

Cells were seeded in 6-well plates at 2 × 10^5^ cells/well 1 day before treatment. Compound C was added to cell cultures as contiguous concentrations from 10 μm with 2-fold dilutions. One week later, cells were harvested and counted using a hemocytometer. The highest concentrations of Compound C that had no apparent toxicity on cell growth were applied to cell proliferation assays or clone formation assays.

To examine the effect of PLX4720 on cellular proliferation, cells were seeded at 5 × 10^4^ cells/well in 6-well plates, and PLX4720 was added to cell cultures as contiguous concentrations from 1 μm with 2-fold dilutions together with or without constant concentrations of Compound C according to the results from the drug toxicity tests. The culture medium with drugs was changed every other day, and 6 day later, cells were harvested and counted as above. The cell growth curves were generated using GraphPad Prism 6.

### Clone formation assay

Cells were seeded in 60-mm dishes at a density of 2 × 10^5^ cells/dish. 24 h later, PLX4720 (0.2 μm) was added to the cell cultures with or without Compound C (0.62 μm). The culture medium was changed every other day, and the concentration of PLX4720 was increased 2-fold every time until it reached 0.8 μm. After 2 weeks of culturing, cells were fixed with 4% paraformaldehyde and stained with crystal violet solution. The clones were counted manually, and the graphs were generated using GraphPad Prism 6.

### Statistical analysis

All statistical analyses in this study were performed using GraphPad InStat (GraphPad Software). Statistical significance was determined by one-sample *t* test in all experiments, and error bars represent standard deviation to show variance between independent experiments.

## Author contributions

J. Yuan, W. H. N., J. Yap, B. C., X. H., and J. H. investigation; M. W. writing-review and editing; J. H. conceptualization; J. H. data curation; J. H. supervision; J. H. methodology; J. H. writing-original draft.

## Footnotes

2 The abbreviations used are:

MEK: mitogen-activated protein kinase/extracellular signal–regulated kinase kinase
ERK: extracellular signal–regulated kinase
NtA: N-terminal acidic
AMPKi: AMP-activated protein kinase inhibitor
CKD: CRAF kinase domain
aa: amino acids
CREB: cAMP response element–binding protein
shRNA: short hairpin RNA
HA: hemagglutinin
Nluc: the N-terminus of firefly luciferase
Cluc: the C-terminus of firefly luciferase
ΔC: deletion of the carboxyl-terminal 14-3-3 binding motif of CRAF
PKA: protein kinase A
ACC: acetyl-CoA carboxylase.
